# Increasing Understanding in Children of Depressed Parents: Predictors and Moderators of Intervention Response

**DOI:** 10.1155/2015/347971

**Published:** 2015-08-19

**Authors:** Tracy R. G. Gladstone, Peter W. Forbes, Anne Diehl, William R. Beardslee

**Affiliations:** ^1^Wellesley Centers for Women, Wellesley College, 106 Central Street, Wellesley, MA 02481, USA; ^2^Clinical Research Center, Boston Children's Hospital, 300 Longwood Avenue, Boston, MA 02115, USA; ^3^Johns Hopkins Bloomberg School of Public Health, 615 North Wolfe Street, Baltimore, MD 21205, USA; ^4^Department of Psychiatry, Boston Children's Hospital, 21 Autumn Street, Boston, MA 02215, USA

## Abstract

We evaluated predictors and moderators of differential response to two family-based depression prevention programs for families with a depressed parent: a clinician-facilitated intervention and a lecture group intervention. Individual and family level variables were examined using regression analyses with generalized estimating equations. For the outcome of child understanding of depression, parental changes in child-related behaviors and attitudes predicted greater child understanding (*p* < 0.001). For the parent outcome of behavior and attitude change, across intervention conditions, younger parent age (*p* < 0.05), female parent gender (*p* < 0.01), more chronic and severe parental depression history (*p* < 0.05), lower SES (*p* < 0.05), and single-parent status (*p* < 0.05) were associated with better outcomes across conditions. Effect sizes were moderate, ranging from 0.4 to 0.7 SD. Family and marital functioning were not found to be predictors of any outcomes. When both parents were depressed at baseline, there was no difference in the clinician- versus lecture-based approach, and when only the father was depressed, families reported more changes with the clinician condition than with the lecture condition (*p* < 0.05). Findings from this study can help identify intervention strategies that are appropriate for different types of at-risk individuals and families.

## 1. Introduction

Depressive illness in youth is a serious public health problem. Depression is the leading cause of disability-related disease burden among 15–44 year olds in developed nations and is associated with lasting negative effects on achievement, physical health, and interpersonal relationships, as well as increased risk of substance abuse and suicide [1–4]. According to the National Comorbidity Survey, 15% of adolescents will have had a depressive disorder by the time they are 18 years old [5]. Although efficacious treatments for youth depression are available, such treatments only successfully treat about 50–60% of cases, even under controlled research conditions [6, 7]. In addition, adolescents are unlikely to seek out treatment, with only about one-quarter to one-third of individuals with depression being involved in any type of treatment, ranging from counseling to medications [8].

Given the high prevalence of pediatric depression, the significant associated impairment, and the difficulty in treating depression once it has developed, efforts to prevent adolescent depression are warranted. A salient target for depression prevention efforts includes children of depressed parents, as parental depressive illness is one of the most potent risk factors for child major depressive disorder (MDD), and children of depressed parents are at a two- to fourfold risk of developing a depressive disorder, compared to children without a depressed parent [9]. A number of mechanisms have been implicated in the transmission of depression from parent to child, including genetic, epigenetic, neuroregulatory, environmental, and parental factors, such as parental child-related behaviors and attitudes [10]. In addition to increasing the risk of child depression, family factors also may maintain depression in youth and affect child response to intervention [11–14].

A logical approach to depression prevention in families with depressed parents is to use a family-based intervention. Avenevoli and Merikangas argue that family-based interventions for families with ill parents are indicated, because parental psychopathology is associated with general dysfunction in the parental/family environment, and changing the environment of at-risk children may lower their risk for depression [15]. Moreover, if children of depressed parents are treated individually but remain in a high-risk environment, then it is possible that they will not benefit fully from the intervention. Interventions with parental involvement may enhance the benefits of adolescent interventions, and family change can enhance resilience in teens [6, 16–18].

For more than 15 years, the Preventive Intervention Project has used a family-based approach to prevent the onset of depression in early adolescents who have no history of depression but are at risk for developing the disorder as a function of having a depressed parent [19–21]. Our approach targets decreasing risk factors associated with parental mood disorder (e.g., family conflict and lack of understanding of parental depression) and increasing factors demonstrated to be protective against the effect of parental illness (i.e., building child resilience by increasing involvement in outside activities, supportive relationships, and understanding of the role of a parent's depression) [22, 23].

Our group developed and tested two family-based preventive interventions for families with parental depression in a randomized trial: a brief clinician-facilitated approach and informational parent lectures. Both interventions provided psychoeducation about parental depression, addressed the negative experiences of individuals in families with a depressed parent (e.g., poor communication, misunderstanding, and feelings of guilt and blame), and provided information regarding ways of enhancing protective factors in children [22]. The lecture intervention consisted of two meetings delivered in a group format without children present, and there was no active attempt to link the psychoeducational material to each family's experiences. The clinician-facilitated intervention included meetings between the professional and the parents and with each child individually, to discuss their understanding and experiences, and a parent-led family meeting to discuss depression in the family and brainstorm ways to enhance the child's support networks and activities outside home.

We contrasted these two active prevention programs and examined preventive effects over several years. Both interventions produced sustained effects 4.5 years after enrollment [19, 22]. Parents in the clinician-based intervention reported significantly more positive changes in their child-related behaviors and attitudes, and their children reported significantly greater understanding of parental disorder than children in the lecture condition. In both groups, child and parent family functioning increased, and child internalizing symptoms decreased, with no significant between-group differences.

An important direction in clinical research is to understand the predictors and moderators of intervention response, so that programs can be targeted to the populations most likely to benefit. We established that both of our prevention strategies are beneficial to children and to families. However, some individuals and families did not respond as well as others. In an effort to maximize response for all participants, we seek to identify the child, parental, and family (i.e., both parents combined, for households with two parents) characteristics that predict or moderate differential child and parental response to each intervention, so that we may tailor our intervention to our participants and optimize their results.

## 2. Materials and Methods

### 2.1. Participants

As described elsewhere, 105 families were randomized to either the lecture or clinician-facilitated condition. Dual- and single-parent families were eligible to participate if they had (1) at least one child 8–15 years of age and (2) at least one parent who had experienced an episode of mood disorder in the 18 months before contact [19, 22]. At the time of recruitment, exclusion criteria included serious current parental substance abuse or dependence, current parental schizophrenia, current severe marital crisis, or other life crises (e.g., hospitalization) that would prevent the family from focusing on the future. Individual treatment of either or both parents was not an exclusion criterion, as we believed it was important for adults to have treatment for managing their mood disorder. However, families currently in marital or family therapy more often than twice per month were excluded, as our family-based prevention approach was best evaluated in the absence of major ongoing family treatment. Youngsters were excluded if their parents reported that they had ever been diagnosed with a mood disorder or were in regular psychotherapy for a mood disorder, but they were not excluded if they had or were being treated for other diagnoses (e.g., learning disabilities and attentional problems). Written informed consent was obtained from both parents and children after the assessment and intervention procedures had been explained fully.

The sample composition has been published previously [22]. Briefly, at baseline, the sample consisted of 105 families including 190 parents and 138 children [22]. Children were 56% male (*n* = 78) and averaged 11.6 years of age. Most parents were white (*n* = 177, 93%) and in their 40s at baseline (*n* = 120, 63%). At baseline, 69 of 190 parents (36%) had depression that had remitted at least 18 months prior to baseline, 67 (35%) had no diagnosed history of depression, and 54 (28%) were currently in a depressive episode (34 (18% of total sample) of whom were poorly functioning and 20 (11% of the total sample) of whom were functioning adequately). Of the 105 families, 85 were led by two heterosexual parents, and 20 were led by female single parents. In our sample, 51 families (49%) had only mothers with active depression at baseline, 32 families (30%) had no active depression in either parent at baseline, 15 families (14%) had only fathers in active depression at baseline, and seven families (7%) had both parents in active depression at baseline. There were no significant differences in baseline demographic variables or psychopathology between the intervention groups on parent or child variables.

### 2.2. Intervention

Our psychoeducational preventive interventions were designed to be used widely in public health settings and hence to be compatible with the customs of pediatricians and family practitioners. The two preventive interventions were clearly specified in manuals. In both interventions, parents were assured that they were not to blame for their depression, that they and their children are separate individuals, and that many children of depressed parents are resilient and do quite well. Both interventions focused on the reduction of individual and familial risk factors over time, as well as on the development of protective factors in adolescents through change in parental attitudes and behaviors. Parents and families were encouraged to share their experiences of the illness with each other. The interventions targeted risk factors that are modifiable (e.g., marital communication and parenting practices) and attempted to address the psychosocial domains that have been linked to the transmission of disorder from parent to child (e.g., parental discord). We designed the interventions so that changes in parental behavior would foster resilient behaviors in children.

The clinician-facilitated intervention consisted of 6 to 11 sessions and included separate meetings with parents and children, family meetings, and telephone contacts or refresher meetings at 6-to-9-month intervals. The core elements of the clinician-facilitated intervention were as follows: (1) assessing all family members; (2) presenting psychoeducational material about mood disorders and about risks and resilience in children; (3) linking the psychoeducational material to the family's life experience; (4) decreasing feelings of guilt and blame in children; and (5) helping the children to develop relationships both within and outside of the family to facilitate their independent functioning in school and in activities outside of home. Thus, in a family meeting, a clinician defined for family members the basic signs and symptoms associated with mood disorder and explored with parents and children family experiences that reflect parental mood disorder. In addition, the clinician encouraged parents to assure children that they were not to blame for parental illness and that they were not able to influence the chronicity or severity of episodes. Finally, the clinician worked with parents to encourage children to pursue interests, relationships, and activities outside of home. Designed to help parents come to a shared understanding of the illness that was then presented to the children in a family meeting, an explicit goal of the clinician-facilitated intervention was to foster families' self-understanding of the illness experience.

The lecture condition consisted of 2 separate meetings delivered in a group format without children present. We hypothesized that children in this intervention program would benefit indirectly from changes made by parents as a result of participation. Although family discussion was encouraged and the psychoeducational material presented mirrored that presented in the clinician-facilitated condition, there was no attempt in the lecture condition to link the cognitive material presented to specific families' individual illness experiences. As in the clinician-facilitated condition, mood disorders were presented in the context of family experience, and parents were encouraged to talk to their children about parental illness. However, in the lecture condition, parents had to decide whether or not to initiate such conversations with their children.

### 2.3. Selection of Potential Moderators

Potential predictor and moderator variables were identified based on a literature review of risk and prevention studies. For example, through Peisah and colleagues and depression risk studies referenced in this work [24–27], we identified parental depression chronicity, parental comorbid anxiety, depressed parent gender, and marital functioning as variables that have been previously associated with differences in child outcomes. Curry et al. indicated that household income might influence outcomes, as well [28]. We were guided by the variable selection utilized by March and colleagues in the TADS study [29], as this is the largest randomized controlled trial targeting the treatment of depression in youth, as well as by the MTA study by Rieppi et al., the largest treatment study of children and adolescents [30]. We also relied on Horowitz and Garber's meta-analytic review of prevention studies for youth depression, which identified several significant variables that warrant consideration [31]. Overall, we identified child (age, gender, race, baseline affective disorder diagnosis, baseline nonaffective disorder diagnosis, and baseline depression diagnosis), parental (age, gender, race, baseline depression history, depression chronicity, suicidal ideation history, and comorbid anxiety history), and family (family composition (e.g., single- versus dual-parent household), household income, which parent is depressed, and baseline family functioning) variables that might predict or moderate differential child and/or parent response to either intervention program.

### 2.4. Measures

Race, parental age, parent gender, family composition (single- versus dual-parent household), household income, and other demographic information were assessed using a measure developed by Larkin and Hirshfeld [32]. Child psychopathology was assessed through parent and child interviews with the Schedule for Affective Disorders and Schizophrenia for School-Age Children, Epidemiologic Version-Revised (Kiddie-SADS-E-R), Life Time Version [33] at baseline (which was scored using the Diagnostic and Statistical Manual of Mental Disorders, 3rd Ed., Revised (DSM-III-R) [34]), and the Kiddie-Streamlined Longitudinal Interval Continuation Evaluation (KSLICE) [35] at all intervals after baseline. The child outcome measures of child internalizing symptoms and child understanding of parental depression, including their feelings, experiences, and awareness of their parents' mood disorder, were assessed through the Youth Self-Report (YSR) [36] and the Semistructured Child Interview (SCI) [22], respectively.

Parental psychopathology and depression chronicity (dichotomized as either more or fewer than 40 weeks of depressive symptoms) were assessed with the Schedule for Affective Disorders and Schizophrenia-Lifetime Version (SADS-L) [37] at baseline, and parental psychopathology was evaluated with the Streamlined Longitudinal Interval Continuation Evaluation (SLICE) at all subsequent assessments [35]. The Global Assessment Scale (GAS) was used to obtain each parent's worst and current levels of functioning during each assessment interval and for the 18 months prior to enrollment [38]. The Semistructured Interview about the Intervention (SII) was administered to each parent at baseline and at all follow-up points to assess parent-reported changes in eight behaviors, such as time spent talking to their children about depression, and nine different attitudes regarding parenting, family communication, and understanding of parental depression [22].

To assess overall change in family functioning, the Family Relationship Inventory (FRI) was administered at baseline and at each follow-up assessment to both parents and children [39]. A full description of the measures used in this study has been published previously [22].

### 2.5. Data Analytic Strategy

The primary outcomes for this study were (1) child internalizing symptoms (YSR), (2) child understanding of parental depression, and (3) parental changes in child-related behaviors and attitudes. Analysis of the child internalizing outcome used baseline internalizing as a covariate to control for baseline differences. The child understanding of parental depression and the parent behavior and attitude change outcomes had no baseline measure. For the child outcomes, we tested to see whether each outcome differed by a number of child (gender, baseline age (two levels: 8–12 versus 13–16), any baseline affective diagnosis, any baseline nonaffective diagnosis, and any baseline depression diagnosis) and family (household income (two levels: <$65 K versus higher), baseline depression severity of the more symptomatic parent (four levels, explained below), single- versus dual-parent household, and gender of the more symptomatic parent) characteristics. Family characteristics also included three continuous measures: baseline marital functioning, baseline depression (Beck Depression Inventory (BDI)), and the larger of the baseline parent behavior and attitudes scores. The four levels of the baseline depression severity of the more symptomatic parent were as follows: never diagnosed with depression, depression sometime before the 18 months prior to baseline, current depression with adequate functioning (GAS of 65 or higher), and current depression with poor functioning (GAS of 64 or below). These child and family characteristics formed a set of “candidate variables,” each of which was compared with each outcome in a separate analysis at the third (one year after intervention) and sixth (two and a half years after intervention) assessments using the candidate variable as the only independent variable in the model. In addition, we looked to see whether the effect of treatment assignment (clinician versus lecture) interacted with each candidate variable. Investigating this interaction allowed us to assess whether the effect of the candidate variable was similar in the two treatment groups. These models included the candidate variable, treatment group, and an interaction term.

Data from the third and sixth assessments were analyzed separately. The third assessment was the first postintervention assessment where a full complement of measures was collected. The sixth assessment was chosen to assess the degree to which intervention effects persist or change over time. The rationale for looking at the data one assessment at a time was that the effect of intervention might be quite different at the two assessments, which were years apart. In general, we looked at the third assessment for an effect of a single baseline candidate variable on each of the three outcomes and then used the sixth assessment data to see whether the effect persisted, diminished, or increased.

The analysis required a method that accounts for correlation between the responses of siblings and parents within a family. These analyses were done with generalized estimating equations (GEE, Proc Genmod, SAS version 9, SAS Institute, Cary, NC). A moderating effect of the candidate variable was tested using a model that added a candidate variable by intervention group interaction. As these are secondary analyses of these data and the original study was not powered to test for interaction effects between treatment group and subject characteristics, the results here should be interpreted as exploratory. In order to adjust *p* values to reduce chance findings due to multiple comparisons, the False Discovery Rate method was used [40]. This method controls the expected proportion of Type 1 errors within families of similar variables (i.e., child understanding of parental depression and parent changes in behavior and attitudes).

## 3. Results

### 3.1. Changes in Child Outcomes over Time

As previously discussed [19, 22], while children in both intervention groups experienced significant decreases in internalizing symptoms over time (mean (SD): baseline: 10.9 (7.7); T3: 9.6 (7.3); T6: 9.0 (7.3); *p* < 0.001), children in the lecture intervention showed significantly less understanding of parental mood disorder than children in the clinician intervention (mean (SD): T3: clinician: 2.7 (2.2) and lecture: 2.0 (2.1); T6: clinician: 3.3 (1.9) and lecture: 2.7 (2.0); *p* < 0.05).

### 3.2. Changes in Parent Outcome over Time

As reported by Beardslee and colleagues [19, 22], while parents in both intervention groups made changes in child-related behaviors and attitudes, parents in the lecture intervention reported significantly fewer changes than did parents in the clinician intervention (mean (SD): T3: clinician: 6.2 (2.9) and lecture: 3.3 (2.3); T6: clinician: 7.7 (3.2) and lecture: 5.0 (3.0); *p* < 0.0001).

### 3.3. Analysis of Child Outcomes

More parent behavior and attitude changes were significantly (*p* < 0.001) associated with greater child understanding of parental depression. For example, at the third assessment, mean child understanding of parental illness in children whose parents were in the lowest quartile of changes (0–3 changes) was 1.9 (SD: 1.8) compared with a mean understanding of 3.5 (SD: 2.1) in children of parents from the highest quartile (9 or more changes). Child internalizing scores were not associated with any of the candidate variables at either assessment, and, after correcting for multiple comparisons, no significant group interactions emerged for this outcome.

### 3.4. Analysis of the Parent Outcome

Analysis of the parent behavior and attitude changes outcome is presented in Table 1. Mothers reported significantly more behavior and attitude changes than fathers at each assessment (effect sizes: T3: 0.47; T6: 0.48, *p* < 0.01). Fathers averaged approximately one and a third fewer behavior/attitude changes than mothers. The sample, being only 7% nonwhite, was not well powered to detect a race effect; no effects of race were found. Younger parents made significantly more changes than older parents at the third assessment (*p* < 0.05) with the oldest parents averaging two fewer changes (effect size: 0.66) than the youngest parents. At the sixth assessment, the differences were only marginally significant (*p* = 0.07), with the oldest group averaging almost two fewer changes than the youngest parents. Severity of baseline depression was significantly associated with the number of changes made at each assessment such that parents with current or past depression at baseline made two more changes than parents without a history of depression (effect size: 0.6, *p* < 0.05; see Table 1). Parents with a history of suicidal ideation (51% of the sample) made significantly more changes than parents without a history of suicidal ideation (*p* < 0.07 at Time 3; *p* < 0.01 at Time 6; effect size: 0.5), although Time 3 result was marginal after correction for multiple comparisons. Lower-income households averaged about one and a quarter more changes than households with higher incomes (T3: effect size: 0.4, *p* < 0.05 at Time 3; T6: effect size: 0.4, *p* < 0.05). Baseline family functioning was not predictive of parent outcomes.

Twenty of the 105 families were single-parent households. Parents in these families reported roughly two more changes than parents in the 85 dual-parent households, at each assessment (effect sizes: T3: 0.6; T6: 0.7, *p* < 0.05). To test whether or not more reported changes in single-parent households were simply a different way of looking at the gender effect finding from the parent-level analysis, we reran a parent-level analysis controlling simultaneously for single-parent status and gender. Adjusting for the gender effect, we still found a significant effect of more changes in single-parent households than in dual-parent households.

**Figure 1:**
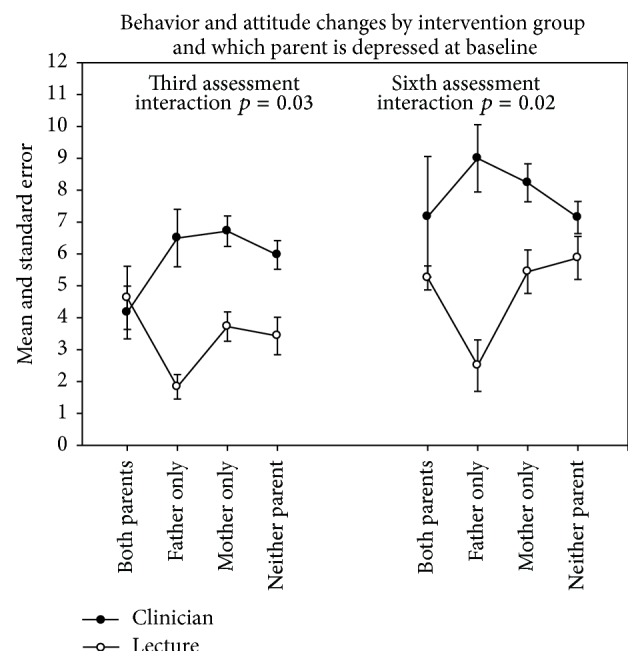
Parental outcome by intervention group and number of parents depressed.

The effect on outcome of which parent is currently depressed varied by intervention at each assessment (Figure 1). When both parents were currently depressed, mean changes were about the same across the two interventions at each assessment. However, when only the father was depressed, fewer changes were made in the lecture intervention, and the most changes were made in the clinician intervention.

## 4. Discussion

This is the first study to look at individual and family level predictors of differential response to a family-based depression prevention program over time. Overall, we were not able to explain changes in children's internalizing symptoms, and we were only able to identify a single variable that predicted children's understanding of parental depression: parental behavior and attitude changes. However, we were able to identify several variables that predict parental intervention response. Specifically, we found that parental gender, age, depression history, depression chronicity, and suicidal ideation history predicted parental changes in child-related behaviors and attitudes (parent baseline comorbid anxiety history was not predictive). That is, mothers, younger parents, and parents with a depression history, with a suicidal ideation history, and with more chronic lifetime depression made significantly more changes than did fathers, older parents, and parents without a history of depression, a history of suicidal ideation, and less chronic lifetime depression.

As Jané-Llopis and colleagues note [41], the influence of gender on effect sizes of depression prevention programs is rarely reported, and some studies have not found child gender to predict or moderate child depression treatment outcomes [28]. Similarly, we found no gender differences in child response to intervention, although our findings are inconsistent with the meta-analytic findings of Stice and colleagues, who found that female gender was associated with larger effect sizes for child depression prevention programs [42]. Among parents, we found that younger participants and female participants made more positive changes in behaviors and attitudes. Little research is available on the influence of parent age or gender as a predictor of family-based intervention response. Overall, our research suggests that our interventions could be adapted for parents of different ages and genders to maximize response of all participants.

Only one of the family level variables we examined predicted increased child response to intervention: more parental behavior and attitude changes. Our findings are inconsistent with the general literature on the connection between marital functioning and children's adaptive functioning [26]. For example, familial and marital discord have been associated with more depression and anxiety disorders in children [43]. However, our findings are consistent with the literature on the relation between parental behaviors and attitudes and offspring well-being [44]. It may be that, in families characterized by strong response to intervention, children are more likely to respond to intervention in kind.

In examining moderators, we found that none of the child (e.g., gender and age) or family (e.g., marital discord) candidate variables moderated the assigned treatment for the outcome of child understanding of parental depression. Likewise, no predictor effects or significant group interactions emerged for the outcome of child internalizing symptoms. These findings are not surprising, as the intervention programs were focused on the parental depression, and the children did not have much exposure to the treatment.

Regarding predictors of parent intervention response, the findings for parent outcome regarding depression history, suicidal ideation history, and depression chronicity are consistent with studies that have indicated that more severely depressed individuals respond better to intervention than less severely depressed participants [45], although some studies have reported the opposite findings [46]. It is possible that parents who have been affected more by depression and suicidal ideation find the information provided in the interventions to be particularly relevant, whereas parents without a depression history do not have as much of a context for the lessons learned and do not find the intervention applicable to them. This may be similar to how universal interventions have smaller effect sizes than selective and indicated interventions [31], because those without the illness do not necessarily find what the intervention offers to be relevant.

Regarding moderators of parent-level response, we found that parental gender, age, and depression severity moderated parental response to intervention. In fact, unlike our results with the children in the sample, we did find a notable gender difference in parent response to intervention, with females reporting more benefit from intervention than males. Similarly, we found notable age differences in parent responses to intervention, but not for child responses. Among parents, younger participants made more positive changes in behaviors and attitudes. Marital and family functioning was not found to moderate the parents' response to either intervention.

For other candidate variables on the family level, we found that family composition (dual- versus single-parent families) and income predicted family response (i.e., combined parental response) to both interventions (family functioning was not predictive). Higher income families did not respond as well as lower-income families, and dual-parent families did not respond as well as single-parent families. That lower-income and single-parent families benefited more from the intervention is a finding that is interesting, as a meta-analysis of parent training studies found significantly poorer outcomes in single and low-income parents [47]. It may be that lower-income families were better able to take advantage of the program, where higher income families had better access to helpful resources prior to enrollment.

When both parents are currently depressed, mean changes were similar for the two interventions, but when only the father was depressed, the lecture condition was not as beneficial as the clinician intervention. It may be that fathers benefit less from group-delivered interventions. This would be consistent with theory-based research that suggests that men who experience gender-role conflict are particularly uncomfortable with psychotherapeutic intervention delivered in a group format [48]. Further analysis is needed to determine why currently depressed fathers did not respond as well to the lecture condition, but future studies may benefit from examining the role of parental gender and baseline depression history or gender-role conflict in differential responses to interventions.

In addition to moderators between our measured variables, it would be very interesting to analyze possible mediators between our three outcome variables. Although we did not explore mediators in this study, there is a noticeable gap in the depression intervention literature on whether or not factors, such as child psychopathology or understanding of parental depression, along with parents' child-related behavior and attitude change, interact with each other when determining treatment response. One example of how these variables could interact is that children who lack an understanding of parental depression or who have a parent with negative child-related behaviors and attitudes may, in fact, be at risk for developing internalizing disorders themselves. The mechanism that underlies the transmission or development of internalizing disorders is crucial to our understanding of how to prevent and treat adolescent depression. This area of research is in need of further investigation.

### 4.1. Limitations

Our analyses offer new insight on parental predictors and moderators of depression preventive intervention response, but there are some limitations. It is important to note that the applicability of these results to other interventions may be limited. More research is needed to see if our findings can be replicated in similar interventions. Our study sample was 97% white, and therefore we cannot say if our findings are applicable across racial and ethnic groups. Also, while our intention was to examine predictors and moderators of differential response, it should be noted that, overall, our parents and children experienced significant benefit from the intervention, and therefore the variables we found to be associated with relatively weaker response may not translate to predictors and moderators of nonresponse. In particular, our findings suggest that the children in the two treatment groups appear to have more similarities than differences, as there were no significant differences between treatment programs.

Lack of a control group makes our findings difficult to interpret. Comparing the group lecture and clinician-facilitated interventions limits our results, as the effects of these interventions have not yet been compared to no-treatment conditions, which would show the efficacy of each intervention. In addition to this, the responses to our two interventions may not have been strong enough to translate into moderator effects, particularly for the child's internalizing symptoms. Finally, the changes from baseline observed in child internalizing may have been partly a response to the intervention and partly a regression to the mean. However, the fact that internalizing scores decreased over these adolescent years, when they might have been expected to increase, suggests that the observed changes were, at least in part, a response to intervention. Further, there is no evidence for change in the variability of this outcome over assessment, suggesting that the observed changes were not the result of moderation over time of extreme baseline levels.

### 4.2. Clinical Implications

The findings of our study suggest that, while parents and children experienced significant benefit from interventions, some children, parents, and families do not respond as well, and individuals and families with certain characteristics respond better to some interventions than others. The findings of this study suggest that a number of child, parent, and family factors should be assessed prior to intervention initiation to optimize the response, paying particular attention to parental age, gender, baseline depression history/chronicity, suicidal ideation history, family income, and family composition (i.e., single- versus dual-parent household).

To ensure more precision in discovering predictors, moderators, and mediators of intervention response in future studies, researchers should include a control group, receiving no form of treatment. As previously discussed, comparing both the lecture condition and the clinician-facilitated interventions individually to a control group would allow us to evaluate the efficacy of each intervention approach. There are also many other variables that can be analyzed as potential moderators and mediators of intervention effects, including parental depression severity and parent-child interaction style.

The outcomes of child understanding of parental depression, child's internalizing symptoms, and parental child-related behavior and attitude change are important variables that need to be further explored. For example, a child's knowledge of depression symptoms and its course with or without treatment are important for future exposures to mental health issues. That is, children who are more aware of what the symptoms of depression are and how treatment can help may be more likely to seek treatment in the future if they begin to experience the symptoms their parents previously displayed. Additionally, there is a gap in the literature involving these types of variables, and it is important that future studies delve into how these outcomes interact with each other and impact a child, parent, or family's response to treatment. Researchers should continue to examine not just predictors, moderators, and possible mediators of optimal response, but also weaker outcomes and nonresponse, to help match participants with the most appropriate interventions available.

## Figures and Tables

**Table 1:** Predictors of parental outcome: parent behavior and attitude change by assessment.

Variable	Subgroup	Third assessment		Sixth assessment	
		Mean # changes (SD)	Significant differences	Mean # changes (SD)	Significant differences
Parent characteristic					
Gender	Father Mother	4.0 (2.8) 5.4 (3.1)	1 < 2^*∗∗*^	5.7 (3.1) 7.0 (3.5)	1 < 2^*∗∗*^
					
Age	30–39 40–49 50–59	5.8 (3.1) 4.5 (3.1) 3.8 (2.2)	1 > 2^*∗*^ 1 > 3^*∗∗*^ 2 = 3		
					
Baseline depression severity	Never Before last 18 months Current but functioning Current + poor functioning	3.7 (2.6) 4.7 (3.1) 5.2 (3.6) 5.7 (2.7)	1 < 2,3, 4^*∗*^	4.9 (2.5) 6.4 (3.2) 7.3 (4.1) 6.9 (3.2)	1 < 2, 3, 4^*∗*^
					
Lifetime depression >40 weeks (yes/no)	Yes No			7.5 (3.3) 6.0 (3.4)	1 > 2^*∗*^
					
Any history of suicidal ideation	Yes No			7.3 (3.4) 5.7 (3.1)	1 > 2^*∗∗*^

Family characteristic					
Household income (2 levels)	To $65K Higher	5.6 (3.3) 4.3 (2.6)	1 > 2^*∗*^	7.2 (3.7) 6.0 (3.0)	1 > 2^*∗*^
					
Single-parent household	Yes No	6.4 (2.7) 4.6 (3.0)	1 > 2^*∗*^	8.4 (2.4) 6.1 (3.0)	1 > 2^*∗*^

^*∗*^ *p* < 0.05; ^*∗∗*^ *p* < 0.01.
